# Treatment of recurrent mandibular ameloblastoma

**DOI:** 10.3892/etm.2013.1165

**Published:** 2013-06-18

**Authors:** PEDRO INFANTE-COSSIO, VICTORIA PRATS-GOLCZER, LUIS-MIGUEL GONZALEZ-PEREZ, RODOLFO BELMONTE-CARO, RAFAEL MARTINEZ-DE-FUENTES, EUSEBIO TORRES-CARRANZA, PURIFICACION GACTO-SANCHEZ, TOMAS GOMEZ-CIA

**Affiliations:** 1 Department of Oral and Maxillofacial Surgery, Virgen del Rocio University Hospital, Spain; 2 Department of Prosthodontics, Faculty of Dentistry, University of Seville, Spain; 3 Department of Plastic and Reconstructive Surgery, Virgen del Rocio University Hospital, Seville, Spain

**Keywords:** recurrent ameloblastoma, odontogenic tumors, jaw surgery, jaw reconstruction, dental implants

## Abstract

Ameloblastoma is a locally invasive benign odontogenic tumor with a high rate of recurrence in the long term. The authors conducted a retrospective study of patients with mandibular ameloblastoma in order to evaluate recurrent ameloblastoma management. The study included data from 31 patients over a period of 10 years. Data collected included age, gender, tumor location, histological findings, initial treatment, number of recurrences and year of onset, type of treatment of recurrence, reconstruction and follow-up. Recurrences were detected in nine patients (29%). Tumor recurrences appeared at 32 months on average following the initial surgical procedure. Recurrences were associated mainly to inadequate initial therapeutic approach and were treated by bone resection with a safety margin of at least 1 cm beyond the radiographically visible margins. Immediate reconstruction of bone defects was performed with grafts or free flaps.

## Introduction

Ameloblastoma is an odontogenic epithelial neoplasm that may originate from the enamel organ, remnants of the dental lamina, epithelium of dentigerous cysts, or possibly from the basal cells of the oral mucosa epithelium (1). Although the lesion is histologically benign, this neoplasm behaves as a slow-growing invasive tumor. Usually the tumor remains asymptomatic until it reaches a large enough size to provoke expansion and perforation of the adjacent soft tissue, at which point the patient may perceive its existence (2).

Ameloblastomas account for ∼1% of all jaw tumors and cysts (3). They appear between 30–40 years of age, except in the unicystic variety which usually appear prior to the age of 30 (4). In >80% of cases ameloblastomas present as an intraosseous neoformation in the mandible, particularly in the molar area or the ascending ramus (5). Since 1992 the World Health Organization has accepted three subtypes of benign ameloblastomas: solid/multicystic, unicystic and extraosseous/peripheral (6). The most common subtype is multicystic, representing >80% of cases including the follicular, plexiform, acanthomatous and granular types. Since 2005, two new subtypes have been added to the classification: desmoplastic and mixed (with areas of desmoplastic and solid pattern). The unicystic type also comprises mural, luminal and intraluminal ameloblastoma arising in dentigerous cysts. Diagnosis is based on imaging examinations (panoramic radiography, CT and MRI) and histopathological studies by means of a biopsy. Radiographically, multicystic ameloblastomas usually present as multilocular radiolucent images in ‘soap bubbles’ (7). In more than half of cases associated with impacted teeth, unicystic ameloblastomas appear as well-defined radiolucent images, with a scalloped or lobed edge. Therefore the tumors are visualized around the tooth crown, similar to that observed in dentigerous cysts (6).

Treatment of these neoplasms remains a matter of debate due to their locally aggressive behavior and high rate of recurrence following treatment (8). The therapeutic challenge is to achieve a complete lesion excision with the least possible morbidity. For this purpose the surgeon is required to assess the location, size and subtype of the ameloblastoma, as well as age of the patient. A number of different treatment strategies have been previously reported including local techniques (curettage, enucleation or marsupialization) or radical treatments (marginal or en-bloc segmental resection with safety margins and reconstruction of bone defect) (2,4,8,9–11). Solid/multicystic ameloblastomas have been identified as the most aggressive subtype, with a high recurrence rate following local excision. By contrast, unicystic ameloblastomas are described to have a lower rate of recurrence and enucleation, with curettage potentially being sufficient for their management. Local treatment has an increased risk of recurrence, therefore it may be complemented with further application of Carnoy's solution, cryotherapy or diathermy in order to reduce the recurrence rate (12). Peripheral ameloblastomas occur in the gingiva or alveolar mucosa and usually respond well to local treatment. The purpose of this study was to analyze the therapeutic results obtained from a series of patients with mandibular ameloblastomas and to specifically focus attention on evaluating the surgical management of recurrent ameloblastoma.

## Materials and methods

A retrospective study was performed on 31 patients with mandibular ameloblastomas, treated at the Department of Oral and Maxillofacial Surgery, ‘Virgen del Rocío’ University Hospital of Seville, Spain, between 2000 and 2010. The patients included 17 men and 14 women, aged between 13–82 years at the time of the initial diagnosis (mean age 43.1 years). Patients with at least one positive ameloblastoma biopsy and who were undergoing surgery were included in the present study. Therapeutic modalities were divided into enucleation, curettage, marginal mandibulectomy, segmental mandibulectomy and mandibulectomy with reconstruction. Enucleation (with or without curettage) involves tumoral removal and avoidance of neoplasm spillage. Marginal mandibulectomy consists of an ‘en bloc’ resection of the tumor with a safety margin of 1 cm of the adjacent bone, and sometimes the adjacent periosteum may be invaded by the tumor. Segmental resection suggests a discontinuity defect of the mandible. Reconstructive surgery of the mandible may be performed with a bone graft or a free flap with or without subsequent placement of dental implants. Initial surgical treatment consisted of enucleation and curettage (26 patients), marginal mandibulectomy (4 patients) and segmental mandibulectomy (1 patient).

The data collected included age, gender, tumor location, histological findings, initial treatment, number of recurrences, year of recurrence onset, surgical treatment option, reconstruction and follow-up. Follow-up was performed by routine annual clinical and radiographic examination by panoramic radiograph and CT. Recurrent ameloblastoma was defined as a relapse after a minimum disease free period of 1 year following initial surgery. Due to the small number of patients enrolled, no statistical analysis was performed.

## Results

In the 10-year period studied, 31 patients underwent surgery for mandibular ameloblastomas. This included 17 men and 14 women, aged 13–82 years at the time of the initial diagnosis (mean age 43.1 years). Sixteen of the 31 patients were under 40 years of age at the time of diagnosis. Multicystic pattern predominated in 26 patients (83.9%), and 5 patients (16.1%) exhibited unicystic pattern. Tumors were located in the molar region of the mandibular body in 16 cases (51.6%), 11 cases affected the ramus and angle (35.5%), and in 4 cases, the anterior and premolar areas were involved (12.9%).

Initial surgical treatment involved enucleation and curettage (26 patients), marginal mandibulectomy (4 patients) and segmental mandibulectomy (1 patient). Nine patients (29%) presented tumor recurrence subsequent to the first surgery. In the 31 patients, a total of 40 surgical procedures were performed, including 26 enucleations and curettages (65%), 9 marginal mandibulectomies (22.5%) and 5 segmental mandibulectomies (12.5%). Primary bone reconstruction was performed in 10 cases: five cancellous bone grafts obtained from proximal tibia, three cortical non-vascularized iliac bone grafts, one iliac crest bone graft and one vascularized fibula free flap were harvested. In the oldest patient (82 years of age), the mandibular continuity defect was reconstructed with a single titanium reconstruction plate. In cases where a segmental resection was performed patients showed permanent anesthesia of the lower lip.

**Figure 1.**
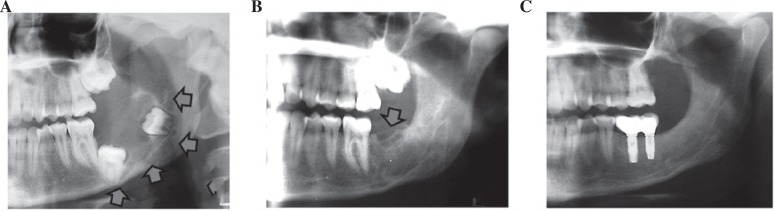
(A) Panoramic radiograph showing a multicystic ameloblastoma in the left mandibular angle associated with the 2nd and 3rd impacted molar (arrows). (B). Recurrent multicystic ameloblastoma located in the mandibular ridge (arrows). (C) Radiographic control after marginal mandibulectomy and reconstruction with cancellous bone graft obtained from proximal tibia, placement of two dental implants and restoration with implant-supported prostheses.

**Figure 2.**
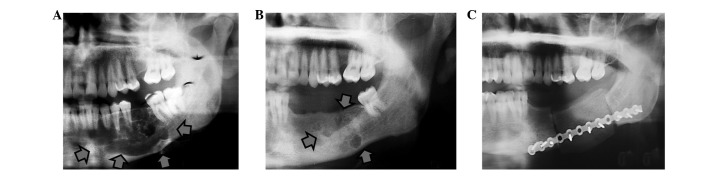
(A) Panoramic radiograph showing a multicystic ameloblatoma in the left mandibular body (arrows). (B) Recurrent ameloblastoma at three sites in the mandibular body (arrows). (C) Radiograph after segmental mandibulectomy and bone reconstruction with iliac crest graft.

**Figure 3.**
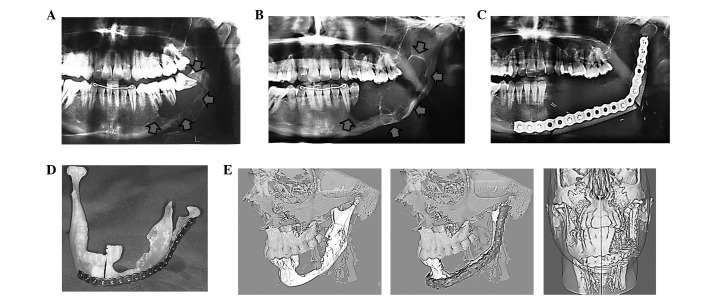
(A) Panoramic radiograph showing a multicystic ameloblastoma in the left mandibular body and angle (arrows). (B) Recurrence of the lesion destroying the body, angle and ramus (arrows). (C) Simulation of tumor resection on a three-dimensional stereolithographic model and pre-bending of the reconstruction plate. (D). Radiograph after segmental mandibulectomy and reconstruction with a fibula free flap. (E) Three-dimensional reconstruction of preoperative planning and outcome after surgical treatment was performed using AYRA software.

The group of patients with recurrent ameloblastomas comprised 2 men and 7 women (mean age of 36.1 years at the time of recurrence). Initial diagnosis was multicystic ameloblastoma in 6 cases and unicystic ameloblastoma in 3 cases. Initial procedures consisted of curettage in 5 cases, and enucleation and curettage in the remaining 4 cases. Recurrences were detected at 32 months on average following initial treatment and were surgically treated by means of marginal mandibulectomies (5 cases) and segmental mandibulectomies (4 cases) (Figs. 1–3). In 6 cases, bone reconstruction was performed primarily (3 patients with cortical iliac crest grafts, 1 patient with cancellous iliac crest bone graft, 1 patient with proximal tibia bone graft and 1 patient with fibula free flap). No further recurrences were observed after the second operation. In 3 patients dental implants were placed posteriorly and implant-supported fixed prosthesis were constructed. Three-dimensional reconstruction of preoperative planning and outcome following surgical treatment was performed using AYRA software (formerly VirSSPA, Andalusian Health Service, Seville, Spain) (Fig. 3E).

## Discussion

Mandibular ameloblastoma management remains a subject of debate. The preferred treatment is surgical excision. However, there is no unanimous consensus with regards to the extent and type of surgery. While the main objective remains to achieve a complete resection, in order to prevent tumor recurrence, the focus of various investigations has been on how to achieve this without performing a disproportionate surgery, for which it is necessary to assess the location, size and type of ameloblastoma, as well as the age of the patient. Currently, conservative local treatment appears to be acceptable in young, growing patients, in order to minimize the psychological impact of an aggressive resection and future functional or growth problems, and in elderly patients to avoid major surgical complications. It is also acceptable in unicystic luminal ameloblastomas, if the tumor has not spread beyond the basement membrane of the cyst, and in those lesions treated without a previously accurate diagnosis (6). Extensive surgical treatment is recommended in large or aggressive ameloblastomas (multicystic) with evidence of cortical bone infiltration or soft tissue extension (13,14).

The sample of 31 ameloblastomas used in the present study reproduce the relatively uniform documented data previously reported with regards to epidemiology, clinical features, treatment and outcomes (4,15–17). The mandibular molar region was the most common location and, in terms of patient age, the group fell within the age ranges reported in the literature. Surgical treatment consisted of conservative treatment in 65% of cases and an en-bloc bone resection (marginal, 22.5% and segmental, 12.5%) in the remaining cases. In accordance with the literature, a more conservative approach to unicyst lesions, which could be treated with simple enucleation and/or curettage, was preferred in young patients (18). In solid and multicystic ameloblastomas we followed the procedure recommended most in the literature, i.e., radical resection including a healthy bone margin of at least 1 cm (19–21).

Recurrence after initial surgical treatment is the result of the infiltrative growth of the ameloblastoma through the adjacent bone, responsible for the local bone cancellous invasion beyond the radiographically visible margins. To a large extent, recurrence is the result of performing an inadequate initial procedure. The recurrence rate should be assessed in a large sample, during a prolonged postoperative period, and varies with regard to location, tumor histology and radicality of the surgical resection. Kim and Jang (22) and Escande *et al* (2) reported an overall recurrence rate of 21.1% and 45%, respectively. In the present study, the overall rate of recurrence was 29%. Hong *et al* (20), in a retrospective analysis of 239 patients with ameloblastomas, reported a recurrence rate of 4.5% after treatment by segmental resection or maxillectomy, 11.6% after marginal resection and 29.3% after conservative treatment (enucleation, curettage and marsupialization), obtaining a statistically significant correlation between method of treatment and recurrence. Attempts have been made to use various markers to differentiate the types of ameloblastoma and prevent recurrences, although this has not yet yielded encouraging results (23). At present, the prognosis of recurrence appears to be associated with the surgical planning prior to evaluation of the histological subtype (1,17,21). In our study, all ameloblastomas that recurred had initially undergone local procedures regardless of type of ameloblastoma.

Radical and aggressive surgery is the preferred option for recurrent ameloblastoma management (4,7,21). This method supports that the mandibular resection should be at least 1–2 cm beyond the radiological limit to ensure that all microlesions are removed (7). In 4 cases a radical treatment option, by means of a segmental mandibulectomy, was selected due to aggressive features (cortical bone perforation, tumor extension, infiltration of the dental nerve, and multicystic type). In all other cases a marginal mandibulectomy was selected, taking into account the preservation of surrounding anatomical structures including dental nerve and basal mandibular cortex.

Mandibular reconstruction is necessary following tumor resection resulting in severe defects of mandibular arch continuity and sacrifice of teeth. Basic reconstruction involves the use of non-vascularized bone grafts together with restoration of lost teeth by means of dental implants and implant-supported prostheses (13,24,25). In 3 cases presenting with <5 cm mandibular segmental defect, reconstruction was achieved using a non-vascularized iliac crest graft. By contrast, in cases where bone resection results in a severe defect of continuity, reconstruction using a micro-vascularized free flap is required (26,27). In the present study, an 8 cm defect involving the body, angle and ramus of the mandible was reconstructed using a fibula free flap. Surgical planning in this particular case was performed using the software VirSSPA AYRA based on the images from CT angiography, which allowed creation of a preoperative stereolithographic model to shape the titanium plate and perform the virtual surgical simulation of bone resection. A template, made using rapid prototyping technology, was used to carry out the fibula osteotomies (28). In marginal mandibulectomies bone regeneration can be expected following preservation of the mandibular basal cortex, particularly in young patients. In 2 patients a cancellous graft, obtained from the proximal tibia and iliac crest respectively, was used to restore the bone defect following a marginal mandibulectomy. In 3 cases, restoration of missing teeth was achieved by secondary dental implant placement in the bone grafts and by using implant-supported prostheses. Previous studies have demonstrated the advantages of implant placement and subsequent restoration with prostheses for the rehabilitation of oral competence, mastication, speech and facial contour in patients with mandibular defects (25,29,30).

In conclusion, ameloblastoma is a benign, locally invasive odontogenic tumor with a high rate of long-term recurrence associated with an inappropriate initial therapeutic approach. When detecting tumor recurrence, the ideal treatment method is bone resection with a safety margin of at least 1 cm beyond the radiographically visible margins. This may be performed by either marginal or segmental mandibulectomy depending on the location and extent of recurrence. Immediate reconstruction of the bone defect with free grafts or flaps, placement of dental implants and rehabilitation with implant-supported prostheses in a second stage can improve jaw function and facial harmony of the patient.
